# Clinical Application of Noninvasive Prenatal Testing for Sex Chromosome Aneuploidies in Central China

**DOI:** 10.3389/fmed.2021.672211

**Published:** 2022-01-26

**Authors:** Ganye Zhao, Peng Dai, Conghui Wang, Lina Liu, Xuechao Zhao, Xiangdong Kong

**Affiliations:** Department of Obstetrics and Gynecology, The Genetics and Prenatal Diagnosis Center, The First Affiliated Hospital of Zhengzhou University, Zhengzhou, China

**Keywords:** noninvasive prenatal testing, sex chromosome aneuploidies, prenatal diagnosis, cell free fetal DNA, genetical counseling

## Abstract

**Background:**

The relatively high incidence and the clinical symptoms of sex chromosome aneuploidies (SCAs) make prenatal screening of SCAs an attractive option for pregnant women. However, limited studies have assessed the clinical performance of noninvasive prenatal testing (NIPT) for screening SCAs. This study was performed to evaluate the clinical performance of NIPT for SCAs in singleton pregnancies in central China.

**Methods:**

Noninvasive prenatal testing was performed using next-generation sequencing. Standard Z-score analysis was used to identify fetal SCAs. NIPT-positive results were confirmed by invasive prenatal diagnosis (IPD).

**Results:**

A total of 42,164 pregnant women with singleton pregnancies were recruited in this study. They were divided into the following five groups with different clinical indications: with ultrasound soft index abnormalities (9.23%, 3,892/42,164); with advanced maternal age (22.14%, 9,336/42,164); with high risk for maternal serum screening (MSS) (18.35%, 7,738/42,164); with an intermediate risk for MSS (26.6%, 11,215/42,164); and with low risk (23.68%, 9,983/42,164). In all, 223 women had a high risk for SCAs by NIPT with a positive rate of 0.53%. There was no significant difference associated with the five groups in the positive rate. Of all of the positive results, 89 were 45,X (39.91%), 38 were 47,XXX (17.04%), 31 were 46,XY,del(X) (13.90%), 50 were 47,XXY (22.42%,), and 15 were 47,XYY (6.73%). Finally, 147 participants (65.92%) chose to undergo IPD, and 47 cases were confirmed. The combined positive predictive value (PPV) of NIPT for SCA was 31.97% (47/147). PPV was high for 47,XYY (100%, 11/11), moderate for 47,XXX (42.86%, 9/21) and 47,XXY (45.45%, 15/33), but low for 45,X (16.13%, 10/62) and 46,XY,del(X) (10%, 2/20). The termination rates of Turner syndrome and 47,XXY syndrome were higher than 47,XXX and 47,XYY syndromes.

**Conclusion:**

In this relatively large cohort, we evaluated the value of NIPT for SCAs. Our data showed that with informed consent and subsequent professional genetical consulting, NIPT can be a useful method to screen SCAs.

## Introduction

The majority of chromosome aneuploidies are lethal and lead to *in utero* death of the fetus. Trisomy 21, trisomy 18, trisomy 13, and sex chromosome aneuploidies (SCAs) including X or Y chromosome abnormality are less severe and present as chromosomal abnormalities in about 0.3% of live births (1). Except for sexual organ dysplasia, SCA may also cause clinical manifestations in other organs, including mental neurological disorders or intellectual disability (2). The most frequently occurring SCAs are 45,X (Turner syndrome); 47,XXX (Triple X syndrome); 47,XXY (Klinefelter syndrome); and 47,XYY (Jacob syndrome) (3). 45,X syndrome is associated with short stature, ovarian dysgenesis, and influences nonverbal abilities, with an incidence of 4:10,000 live births. 47,XXX syndrome is associated with tall stature, mild learning difficulties, and social communication problems, with an incidence of 1:1,000 live births. 47,XXY syndrome is associated with infertility, tall stature, and influences verbal skills and social competence with an incidence of 1:750 live births. 47,XYY syndrome is associated with a high risk of impulsivity and autistic traits, with an incidence of 1:1,000 live births (3). These relatively high incidences of SCAs and their clinical symptoms make prenatal screening of SCAs an attractive option for pregnant women.

There are many methods for screening for trisomy 21, trisomy 18, and trisomy 13, including maternal serum screening (MSS), ultrasound, and noninvasive prenatal testing (NIPT) (4). In contrast, limited methods can be used to screen SCAs, as the clinical features of most SCAs are not detectable during prenatal ultrasound examination because many develop after birth with the exception of structural anomalies more commonly seen in the Turner syndrome (1, 5–7). With the discovery of circulating cell-free fetal DNA (cffDNA) in the blood samples of pregnant women and the advent of massively parallel genomic sequencing (MPS) technology, NIPT has been widely applied for the clinical screening for fetal chromosomal aberrations and has already expanded beyond the three common autosomal trisomies to include SCAs (8, 9).

An increasing number of reports have proven that NIPT is the most accurate screening test for trisomy 21, trisomy 18, and trisomy 13 with high sensitivity and specificity (9–22). However, limited studies have evaluated the accuracy of NIPT for screening SCAs (7, 8, 23). Therefore, we investigated the application of MPS-based NIPT for screening of SCAs among pregnant women in our center in central China. Compared with the results of invasive prenatal diagnosis (IPD) and follow-up, we evaluated the clinical performance of NIPT for detecting fetal SCAs.

## Materials and Methods

### Study Design

Suitable pregnant women were fully informed by the treating doctor of the objectives, significance, accuracy, risk, and limitations of NIPT. All the participants provided written informed consent and chose to undergo NIPT after understanding the purpose of the test and study. Then, post-test clinical genetic counseling was offered to the women with the suspected risk of SCAs by qualified clinical geneticists. Further IPD was carried out for high-risk pregnant women after obtaining informed consent. Finally, we assessed the clinical value of NIPT for screening SCAs from multiple aspects.

### Cohort

The retrospective study was conducted from January 2016 to June 2019 in pregnant women undergoing NIPT at the First Affiliated Hospital of Zhengzhou University, Zhengzhou, Henan Province, Central China. Women were categorized into five groups based on different clinical indications. Ultrasound soft index abnormalities group (including echogenic intracardiac focus, choroid plexus cysts [uni- or bilateral cysts], pyelectasis, thickened nuchal translucency or nuchal fold, hyperechogenic bowel, absent or shortened nasal bone length, single umbilical artery); Advanced maternal age group (aged ≥ 35 years); The high-risk group for MSS (a value of ≥ 1/270 means high risk for trisomy 21 and a value of ≥1/350 means high risk for trisomy 18); intermediate-risk group for MSS (a value from 1/271 to 1/1,000 means intermediate risk for trisomy 21, and a value from 1/351 to 1/1,000 means intermediate risk for trisomy 18); low-risk group (with none of the aforementioned risk factors, women in this group chose NIPT for screening chromosome aberrations because of maternal anxiety).

### Noninvasive Prenatal Testing

Approximately 10 ml of the peripheral blood was collected in a cell-free DNA™ BCT tube (Streck, Omaha, NE, USA), according to protocol of the manufacturer. The collected blood samples were centrifuged for 10 min at 1,600 g and 4°C. Then, the supernatant was centrifuged for 10 min at 16,000 g and 4°C to remove any residual blood cells. Cell-free DNA (cfDNA) was extracted from the plasma using a cfDNA extraction isolation kit (Berry Genomics Corporation, Beijing, China) according to instructions of the manufacturer. The sequencing library was then constructed and purified using an NIPT library prep kit (Berry Genomics Corporation, Beijing, China). A NextSeq CN500 MPS kit (Berry Genomics Corporation, Beijing, China) was used to sequence the sample using the NextSeq CN500 sequencer (Berry Genomics Corporation, Beijing, China). At least 2 million 36-bp clean reads were generated for each sample. Sequencing results were mapped and processed using the data analysis system (Berry Genomics Corporation, Beijing, China). Chromosomes were classified as abnormal if an absolute Z score was greater than 3.

### Invasive Prenatal Diagnosis

Women with positive NIPT results were offered ultrasound-guided amniocentesis. The karyotype analysis of fetal amniotic fluid exfoliated cells was performed with amniocyte culture using Giemsa-banding techniques. For each sample, more than 30 metaphase cells were analyzed at a resolution of 320 G-bands at least. The accuracy of NIPT for SCA was assessed based on the results of fetal chromosomal karyotyping. CNV-seq was performed according to standard procedures. In short, DNA extracted from fetal amniotic fluid or uncultured peripheral blood samples was fragmented. Then, sequencing libraries constructed were sequenced on the Next-Seq CN500 platform. Approximately 5 million raw-sequencing reads with 36 bp in length were generated. The results were analyzed using the previously described algorithms to detect the microdeletions and duplications (24).

### Follow-Up

The positive cases were followed up within 3 months after we sent the reports, which was defined as a prenatal follow-up. A second follow-up was conducted to know the pregnancy outcomes of those cases within 12 months after delivery, which was defined as a postpartum follow-up.

### Statistical Analysis

Statistical Product and Service Solutions (SPSS) software (version 21, IBM Corporation, Armonk, NY, USA) was used for all data analyses. The chi-squared test was used to test the differences of proportions for statistical significance. *P* < 0.05 was considered to indicate statistical significance. Positive predictive value (PPV) was calculated as the number of cases for which the results of NIPT and IPD were concordant.

## Results

### Characteristics of Pregnancies for NIPT

**Figure 1:**
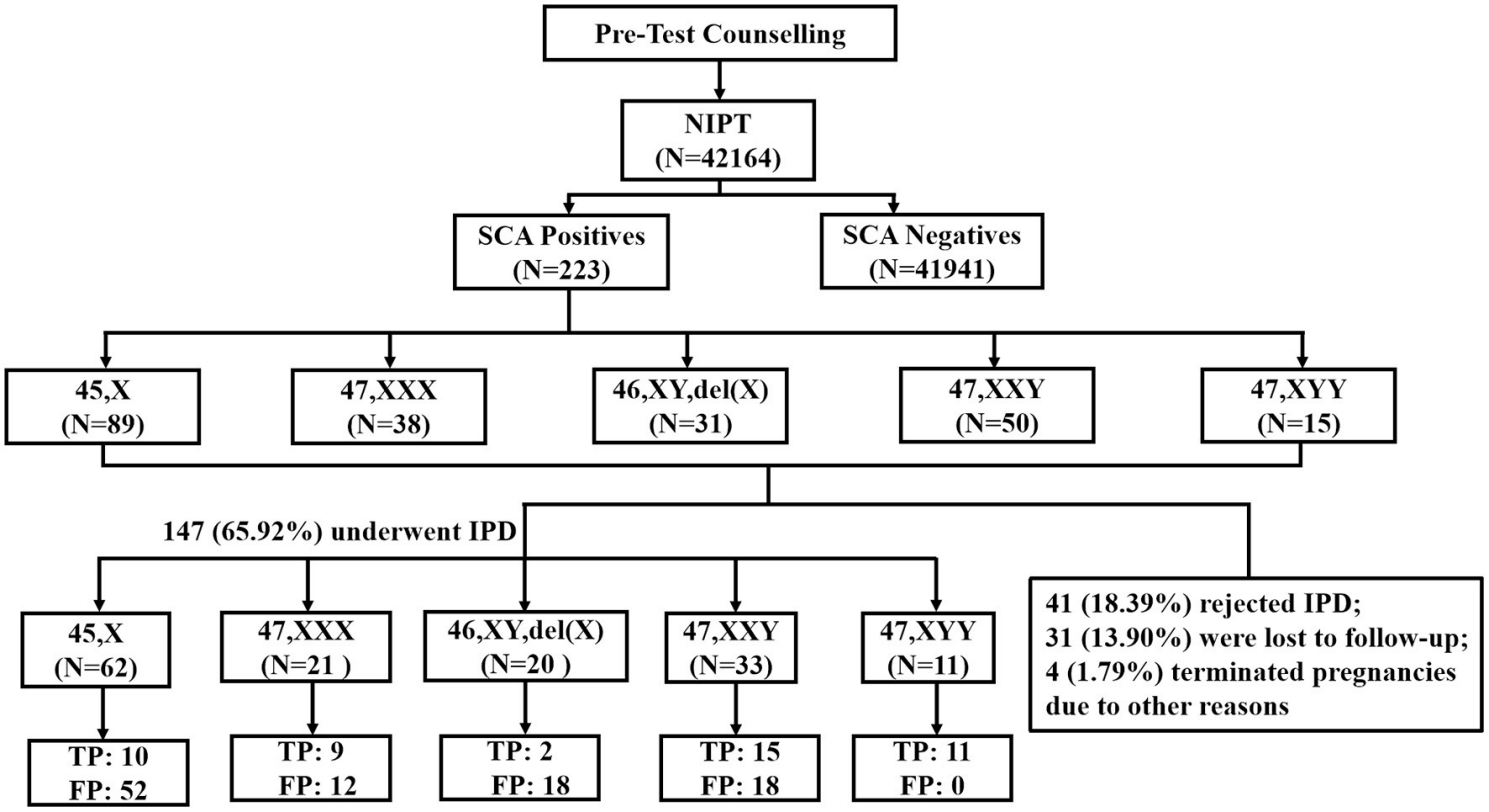
Flowchart of NIPT results for sex chromosome abnormality. SCA, sex chromosome aneuploidy; IPD, invasive prenatal diagnosis; TP, true positive; FP, false positive.

Excluding the samples that could not be detected, 42,164 singleton pregnant women were recruited in this study (Figure 1). The singleton pregnancies included 40,092 (95.09%) natural conceptions and 2,072 (4.91%) assisted conceptions. The majority (75.75%) of pregnant women were at <35 maternal age. The majority (91.64%) of the cohort were at 11-22^+6^ gestational age when NIPT was performed. The mean age of pregnant women was 30.4 ± 5.2 years, and the mean gestational age was 18 ± 3.2 weeks. Of the 42,164 participants, 3,892 (9.23%) showed ultrasound soft index abnormalities; 9,336 (22.14%) were of advanced maternal age; 7,738 (18.35%) were at high risk for MSS; 11,215 (26.6%) were at intermediate risk for MSS; and 9,983 (23.68%) were at low risk (Table 1).

**Table 1:** Characteristics of the study population.

**Indications**	**Number (%)**
Ultrasound soft index abnormalities	3,892 (9.23%)
Advanced maternal age	9,336 (22.14%)
High risk for MSS	7,738 (18.35%)
Intermediate risk for MSS	11,215 (26.6%)
Low risk	9,983 (23.68%)
**Conception**	
Natural conceptions	40,092 (95.09%)
Assisted conceptions	2,072 (4.91%)
**Maternal age (years)**	
<35	31,939 (75.75%)
35–40	8,388 (19.89%)
40–50	1,833 (4.35%)
≥50	4 (0.01%)
**Gestational age at NIPT (weeks)**	
11–22^+6^	38,641 (91.64%)
23–29^+6^	3,050 (7.23%)
30–34^+6^	451 (1.07%)
≥35	22 (0.05%)

*MSS, maternal serum screening*.

### The Results of NIPT

Noninvasive prenatal testing identified 223 positive SCA cases-−45,X (*n* = 89, 39.91%); 47,XXX (*n* = 38, 17.04%); 46,XY,del(X) (*n* = 31, 13.90%), 47,XXY (*n* = 50, 22.42%); and 47,XYY (*n* = 15, 6.73%)—yielding an overall SCA detection rate of 0.53% (Table 2). The mean ± SD maternal age of the 223 women with SCA-positive NIPT results was 30.2 ± 5.3 years, and the mean gestational age at testing was 18.4 ± 2.9 weeks. Of the SCA-positive cases, there were 25 in the ultrasound soft index abnormalities group (positive rate: 0.64%), 43 in the advanced maternal age group (positive rate: 0.46%), 39 in the high-risk for MSS group (positive rate: 0.50%), 61 in the intermediate risk for MSS group (positive rate: 0.54%), and 55 in the low-risk group (positive rate: 0.55%). There was no significant difference associated with these groups (*p* > 0.05) (Table 2).

**Table 2:** Positive rate of NIPT for fetal sex chromosome abnormality in different indications.

	**Positive rate (95%CI)**					
**Indications**	**45,X**	**47,XXX**	**46,XY,del(X)**	**47,XXY**	**47,XYY**	**Total**
Ultrasound soft index abnormalities	0.23% (0.11–0.45%)	0.13% (0.05–0.32%)	0.10% (0.03–0.28%)	0.15% (0.06–0.35%)	0.03% (0.00–0.17%)	0.64% (0.42–0.96%)
	(9/3,892)	(5/3,892)	(4/3,892)	(6/3,892)	(1/3,892)	(25/3,892)
Advanced	0.19% (0.12–0.31%)	0.07% (0.03–0.16%)	0.09% (0.04–0.18%)	0.10% (0.05–0.19%)	0.01% (0.00–0.07%)	0.46% (0.34–0.63%)
maternal age	(18/9,336)	(7/9,336)	(8/9,336)	(9/9,336)	(1/9,336)	(43/9,336)
High risk	0.23% (0.14–0.37%)	0.13% (0.07–0.25%)	0.04% (0.01–0.13%)	0.08% (0.03–0.18%)	0.03% (0.01–0.11%)	0.50% (0.36–0.69%)
For MSS	(18/7,738)	(10/7,738)	(3/7,738)	(6/7,738)	(2/7,738)	(39/7,738)
Intermediate risk	0.21% (0.14–0.32%)	0.09% (0.05–0.17%)	0.08% (0.04–0.16%)	0.10% (0.05–0.18%)	0.06% (0.03–0.13%)	0.54% (0.42–0.70%)
for MSS	(24/11,215)	(10/11,215)	(9/11,215)	(11/11,215)	(7/11,215)	(61/11,215)
Low risk	0.20% (0.13–0.31%)	0.06% (0.02–0.14%)	0.07% (0.03–0.15%)	0.18% (0.11–0.29%)	0.04% (0.01–0.11%)	0.55% (0.42–0.72%)
	(20/9,983)	(6/9,983)	(7/9,983)	(18/9,983)	(4/9,983)	(55/9,983)
Total	0.21% (0.17–0.26%)	0.09% (0.06–0.12%)	0.07% (0.05–0.10%)	0.12% (0.09–0.16%)	0.04% (0.02–0.07%)	0.53% (0.46–0.61%)
	(89/42,164)	(38/42,164)	(31/42,164)	(50/42,164)	(15/42,164)	(223/42,164)

*MSS, maternal serum screening*.

### The Invasive Prenatal Diagnosis Results of Positive NIPT Cases

After the pretest clinical counseling, all 223 (100%) participants acknowledged the risks associated with SCAs in their fetuses. These positive cases were followed up within 3 months after we sent the reports. The prenatal follow-up results showed that of the 223 participants, 41 (18.39%) rejected further prenatal diagnosis; 31 (13.90%) were lost to follow-up; 4 (1.79%) chose to medically terminate their pregnancies due to other reasons including gestation-induced hypertension and structural abnormalities detected during the late trimester (Figure 1). Finally, 147 (65.92%) women underwent IPD and 47 cases were confirmed as SCA-positive (*n* = 10 [45,X]; *n* = 9 [47,XXX]; *n* = 2 [46,XY,del(X)]; *n* = 15 [47,XXY]; and *n* = 11 [47,XYY]), while the remaining 100 cases had normal karyotypes or polymorphism or other chromosomal abnormalities (Figure 1). The combined PPV of NIPT was 31.97% (47/147) for detecting fetal SCAs. The PPV for individual SCA was as follows: 47,XYY (100.00%, 11/11); 47,XXY(45.45%,15/33); 47,XXX (42.86%, 9/21); 45,X(16.13%,10/62); and 46,XY,del(X) (10.00%, 2/20) (Table 3). The false-negative rate for SCAs in our study was unavailable, as most of the affected children do not present obvious symptoms in their childhood and the majority of them had no neonatal karyotype information.

**Table 3:** Performance of NIPT for detecting sex chromosome abnormality.

**SCA**	**True positive**		**False positive**	**PPV (95% CI)**
	**Full**	**Mosaicism**		
45,X	6 (60%)	4 (40%)	52	16.13% (9.00–27.21%)
47,XXX	7 (77.78%)	2 (22.22%)	12	42.86% (24.47–63.46%)
46,XY,del(X)	0 (0%)	2 (100%)	18	10.00% (2.79–30.10%)
47,XXY	14 (93.33%)	1 (6.67%)	18	45.45% (29.84–62.01%)
47,XYY	11 (100%)	0 (0%)	0	100.00% (74.12–100%)
SUM	38 (80.85%)	9 (19.15%)	100	31.97% (24.97–39.89%)

*SCA, sex chromosome abnormality; PPV, positive predictive value*.

### Detection of Sex Chromosome Mosaicism

In the 147 high-risk cases who underwent IPD, nine mosaicism cases were confirmed (Table 3). For all the mosaicism cases, the mean ± SD maternal age was 29.6 ± 7.3 years, the mean gestational age at testing was 17.2 ± 1.7 weeks. The proportion of mosaic anomalies was between 14.00% and 83.33% (Table 4). Cases 1, 4, 6, 7, 8, and 9 chose to terminate the pregnancy. Cases 2 and 5 chose to continue the pregnancy. These two children are now developing normally at the time we followed up. Case 3 was lost to the second postpartum follow-up (Table 4).

**Table 4:** Detection of sex chromosome mosaicism.

**Case**	**Maternal age (years)**	**Gestational age (weeks)**	**NIPT suggested results**	**Results of prenatal diagnosis**	**Proportion of mosaic anomalies**	**Follow-up results**
Case 1	22	13^+2^	45,X	45,X[27]/46,XX[23]	54.00%	Terminated
Case 2	25	19^+0^	45,X	45,X[14]/46,XX[16]	46.67%	Born
Case 3	25	18^+3^	45,X	45,X[5]/46,XX[25]	16.67%	NA
Case 4	22	17^+5^	45,X	45,X,inv(9)(p11q13)[10]/46,XX,inv(9)(p11q13)[20]	33.33%	Terminated
Case 5	41	17^+0^	47,XXX	47,XXX[7]/46,XX[23]	23.33%	Born
Case 6	40	18^+0^	47,XXX	47,XXX[25]/46,XX[5]	83.33%	Terminated
Case 7	30	17^+5^	46,XY,del(X)	45,X[23]/46,X,Yqh-[17]	57.50%	Terminated
Case 8	27	17^+0^	46,XY,del(X)	45,X[7]/46,XY[43]	14.00%	Terminated
Case 9	34	16^+3^	47,XXY	47,XXY[35]/46,XX[10]	77.78%	Terminated

*NA, not available*.

### Cases With Incidental Findings

**Figure 2:**
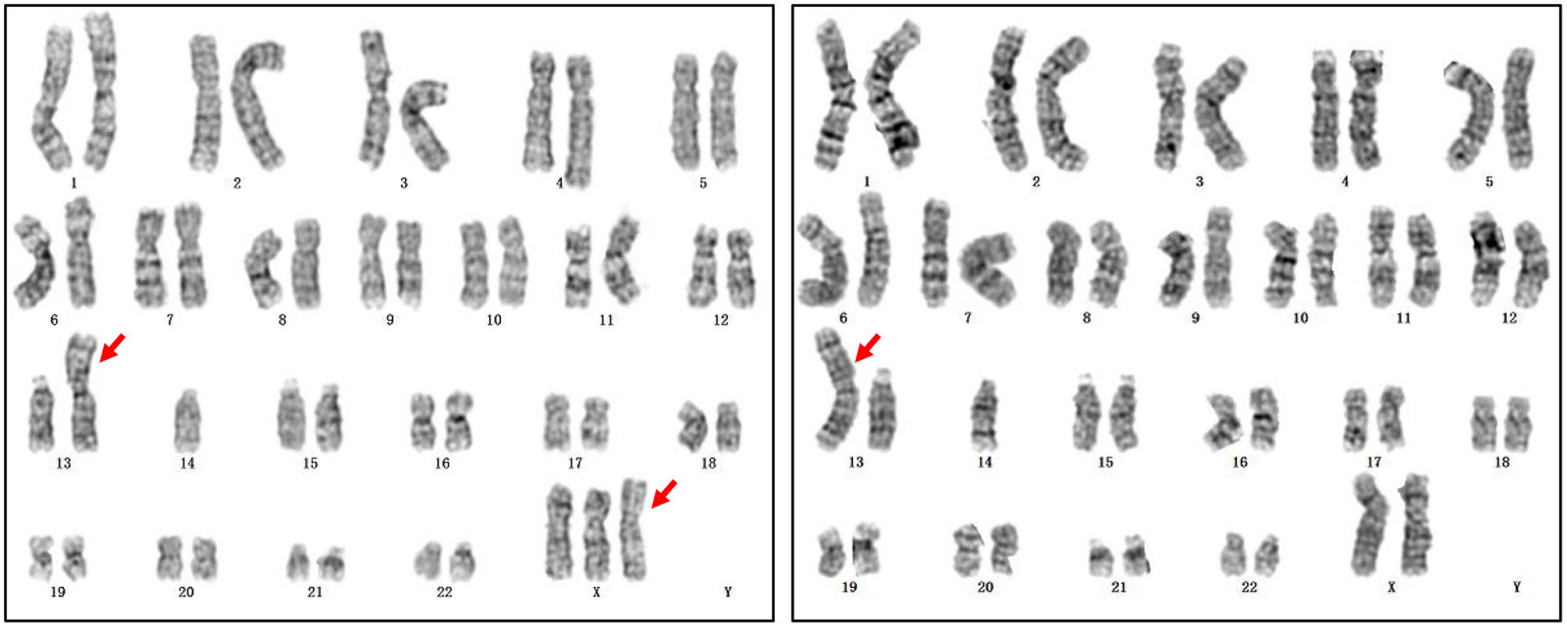
The maternal karyotype of case 11. The karyotype was mos 46,XXX,rob(13;14)(q10;q10)[31]/45,XX,rob(13;14)(q10;q10)[19].

In addition, prenatal diagnosis and maternal chromosome analysis confirmed eight cases with incidental findings including maternal chromosome abnormality (case 10 and case 11), chromosome balanced translocation (case 12), chromosome polymorphism (case 13 and case 14), and chromosome copy number variation (case 15, case 16, and case 17). NIPT suggested that case 10 was 47,XXX, while it was confirmed to be false positive by the prenatal diagnosis. However, the maternal karyotype was 47,XXX, which led to the discordant result. For case 11, the karyotype was suspected to be 47,XXY, but prenatal diagnosis results showed that the karyotype of the fetus was Robertsonian translocation with no sex chromosome aberration. However, the maternal karyotype of the pregnant woman was Robertsonian translocation with mos 46,XXX[31]/45, XX[19] (Figure 2), the false-positive NIPT results were because of the background of maternal chromosome mosaicism. For case 15, the fetus had a deletion of 7.56 Mb at chromosome 18, which was *de novo* deletion as both of the parents were normal. The fetus was more likely to develop into 18p partial deletion syndrome in the future (25). For cases 16 and 17, they all had copy number variation with no pathogenicity. For case 16, the microdeletion of chromosome 16 was inherited from his mother with no abnormal clinical manifestations. The follow-up results showed that cases 10, 11, 12, 13, 14, 16, and 17 chose to continue the pregnancy and are now developing normally at the time we followed up. Case 15 chose to terminate the pregnancy (Table 5).

**Table 5:** Cases with incidental findings.

**Case**	**Maternal age (years)**	**Gestational age (weeks)**	**NIPT suggested results**	**Karyotype of the fetus**	**CNV-seq results of the fetus**	**Maternal karyotype**	**Follow-up results**
Case 10	33	12^+1^	47,XXX	46,XX	NA	47,XXX	Born
Case 11	30	17^+1^	47,XXY	45,XY,rob(13;14) (q10;q10)	seq[hg19]46,XY	46,XXX,rob(13;14) (q10;q10)[31]/45,XX, rob(13;14)(q10;q10)[19]	Born
Case 12	34	20^+1^	45,X	46,XX,t(1;19) (q42;p13)	seq[hg19]46,XX	NA	Born
Case 13	27	18^+2^	45,X	46,XX,1qh+	seq[hg19]46,XX	NA	Born
Case 14	32	25^+3^	47,XXY	46,XY,13pss	NA	NA	Born
Case 15	20	19^+1^	45,X	46,XX	seq[hg19]18p11.32p11.23 (120000-7,680,000) ×1)	46,XX	Terminated
Case 16	25	20^+0^	47,XXY	46,XY	seq[hg19]16p12.2 (21951415-22415560) ×1	seq[hg19]16p12.2 (21951415-22415560) ×1	Born
Case 17	35	16^+5^	46,XY,del(X)	46,XY	seq[hg19]16p11.2(29640000-30200000) ×1	NA	Born

*NA, not available*.

### Postpartum Follow-Up Results of the Rejected Groups and the True Positive Cases

We followed the pregnancy outcomes of the rejected groups and the confirmed true positive cases within 12 months after delivery. Of the 41 cases without IPD, 24 (58.54%) had a live birth and no visible abnormality, and 4 (9.76%) chose to terminate the pregnancies. A total of 13 (31.70%) cases lost the second follow-up. Of the 47 cases with true-positive SCA, 14 (29.79%) had a live birth and no visible abnormality at the time of follow-up; 21 (44.68%) chose to terminate the pregnancies, and 1 (2.13%) underwent spontaneous abortion; 11 lost the second follow-up (Table 6).

**Table 6:** Parental decision and pregnancy outcomes for the confirmed positive cases and cases without the invasive prenatal diagnosis.

**NIPT results**	**Rejected diagnosis**			**Confirmed positive cases**			
	**Terminated**	**Born**	**NA**	**Terminated**	**Born**	**Spontaneous abortion**	**NA**
45,X	3 (25.00%)	6 (50.00%)	3 (25.00%)	6 (60.00%)	1 (10.00%)	1 (10.00%)	2 (20.00%)
47,XXX	0 (0.00%)	7 (70.00%)	3 (30.00%)	2 (22.22%)	5 (55.56%)	0 (0.00%)	2 (22.22%)
46,XY,del(X)	0 (0.00%)	5 (83.33%)	1 (16.67%)	2 (100%)	0 (0.00%)	0 (0.00%)	0 (0.00%)
47,XXY	1 (9.10%)	5 (45.45%)	5 (45.45%)	9 (60.00%)	3 (20.00%)	0 (0.00%)	3 (20.00%)
47,XYY	0 (0.00%)	1 (50.00%)	1 (50.00%)	2 (18.18%)	5 (45.45%)	0 (0.00%)	4 (36.37%)
SUM	4 (9.76%)	24 (58.54%)	13 (31.70%)	21 (44.68%)	14 (29.79%)	1 (2.13%)	11 (23.40%)

*NA, not available*.

## Discussion

Noninvasive prenatal testing has high sensitivity and specificity, and it is widely used worldwide for screening trisomy 21, trisomy 18, and trisomy 13. As for SCAs, the detection accuracy varies among different research groups. The positive rate of SCA in our study was 0.53%. There was no significant difference associated with the five groups in the positive rate, which means there was no clear correlation between SCAs and these indications including ultrasound abnormalities, maternal age, and MSS. Of the 223 SCA-positive cases, 65.92% chose to undergo IPD, which was consistent with previously reported data (8, 22, 26). The IPD rates of SCA-positive cases were lower than that of trisomy 21/18/13 (data not shown), which was because SCAs are less severe and there exist some effective medical intervention methods for SCAs. This is consistent with the study of another research group (22). Most of the people who rejected prenatal diagnosis preferred to choose to give birth to these fetuses (Table 6).

The overall PPV for SCAs in our study was 31.97%. The PPV for 47,XYY syndrome, 47,XXY syndrome, 47,XXX syndrome, 45,X syndrome, and 46,XY,del(X) were 100.00%, 45.45%, 42.86%, 16.13%, and 10.00%, respectively, which were similar to a previous report (8). Although the performance of PPV for SCAs was different in different studies because of varied sample sizes and the sequencing platform used, NIPT performed better in predicting sex chromosome trisomy than monosomy X in most studies (8, 21, 26).

In our study, the maternal peripheral blood chromosomes were detected for only a few cases according to their own accord. The maternal peripheral blood chromosomes of case 10 were proven to have triple X and the maternal karyotype of case 11 was mos 46,XXX,rob(13;14)(q10;q10)[31]/45,XX,rob(13;14)(q10;q10)[19], which led to discordant results (Table 5). Maternal SCA (full or mosaic) is a common and important cause of discordant SCA (1, 27) because the majority of plasma cfDNA of pregnant women are derived primarily from cellular apoptosis of the hematopoietic system (28). Thus, determining the maternal karyotype would increase the accuracy of NIPT results for SCA (1, 29). However, as the associated costs would increase, it is difficult to make maternal karyotyping routinely available during pregnancy check-up visits. On the other hand, once the maternal copy number variation is accidentally found, it would cause unnecessary anxiety to pregnant women in the future, even if the copy number variation is benign.

As circulating cffDNA has been shown to mainly originate from the cytotrophoblast cells (30), confined placental mosaicism or placental mosaicism are other significant factors for the discordant results, such as false positive and false negative results, which occur in about 2% of all pregnancies (31). It is very difficult to prenatally confirm confined placental mosaicism in a pregnant woman. However, one study implemented a novel analytical approach that could detect the presence and estimate the degree of confined placental mosaicism, with accurate prediction in 90% of the cases (32). If this method can be further optimized, the identification of potential mosaicism would greatly improve clinical guidance during pregnancy.

The poorer performance in screening monosomy X could partially be due to a highly variable amplification of chromosome X because of its lower guanosine-cytosine content (33). Although not detected in this study, low levels of mosaicism for monosomy-X can be present in apparently healthy women (34), i.e., the maternal mosaicism for 45,X relatively more frequently influences the effectiveness in predicting the fetal 45,X karyotype. Nearly half of the true positive cases of 45,X were mosaicism in this study (Table 3). Another potential reason could be a vanishing twin affected by monosomy-X.

Fetal mosaicism is very common among SCAs, compared with the three common autosomal aneuploidies. About 15% of cases of 47,XXY, 10% of cases of 47,XXX, and up to 50% of cases of monosomy X are mosaic, which may lead to a nonuniform distribution of sex chromosomes influencing the accuracy of classifying such anomalies (35). As in our study, 19.15% (9/47) of all the true positive results were mosaicism. Furthermore, 40% (4/10) cases of 45,X; 22.22% (2/9) cases of 47,XXX; 100% (2/2) cases of 46,XY,del(X); and 6.67% (1/15) cases of 47,XXY were mosaicism. No mosaicism was found in 47,XYY. In summary, our study showed good performance in detecting the mosaicism.

The necessity of screening SCA by NIPT is debatable with respect to their relatively mild phenotypes of SCA compared with trisomy 21/18/13. Hence, it is more complicated in counseling SCAs after diagnosis. Clinicians should exercise more patience and be empathetic when explaining the possible symptoms and prognosis to soothe the possibility of the parental anxiety. On the other hand, the affected children could be benefitted from the prenatal screening and diagnosis of SCA, providing an opportunity for early intervention and comprehensive postnatal management including hormone therapy and targeted surveillance, as hormonal treatment could induce puberty in Turner syndrome and androgen supplementation is also beneficial in Klinefelter syndrome (3, 36). The termination rates were different among different types of SCAs. The termination rates of Turner syndrome and 47,XXY syndrome were higher than 47,XXX syndrome and 47,XYY syndrome, which was similar to the previous reports (26, 37). The difference may be caused by the clinical manifestations related to high risks of infertility and lack of secondary sexual characteristics (26). Besides, the decision to terminate or continue pregnancy may also be related to the level of prenatal genetic counseling, the cognition level of the pregnant woman, economic situation, and cultural factors (37). Therefore, it is very important to provide professional and comprehensive genetical counseling for women with positive NIPT results for SCAs (38).

Our study adds further support for NIPT as an effective screening method for the detection of SCAs in pregnant women with different kinds of clinical indications in a large cohort of singleton pregnancies in central China. However, there are a few limitations to our study. First, the sensitivity, specificity, and negative predictive value were not calculated due to the absence of neonatal karyotype information, as most of the affected individuals do not present anomalies in the neonatal period. Second, we could not obtain all of the information about maternal or placental mosaicism contributing to the false positive rate. Third, though counseling clinicians of the participants are typically contacted in the prenatal and postnatal phases to obtain diagnostic results, many are still lost, which resulted in incomplete confirmation of NIPT positive results by karyotyping and a lack of the postnatal data.

In conclusion, our data with a relatively large sample size showed that NIPT can be used to screen SCAs with similar performance as in the previous reports (8, 26), providing more comprehensive and reliable reference information in the genetic consulting for SCAs. After adequately informing pregnant women of the availability of the SCA screening along with the ability to provide accurate, balanced, and up-to-date information for subsequent genetic counseling when a fetus is diagnosed with SCA, pregnant women can benefit from NIPT-based SCA screening.

## Data Availability Statement

The original contributions presented in the study are included in the article, further inquiries can be directed to the corresponding author.

## Ethics Statement

This study was conducted in accordance with the Declaration of Helsinki and was approved by the Ethics Committee of The First Affiliated Hospital of Zhengzhou University (2018–016). All participants signed the written informed consent.

## Author Contributions

XK designed the project and edited the manuscript. GZ was involved in project management, writing, performing the experiment, and data analysis. PD, CW, LL, and XZ performed the experiment. All the authors contributed to the article and approved the submitted version.

## Funding

This study was supported by grants from the Science and Technology Research Program of Henan Province [Grant No. 202102310391], the Youth Innovation Fund of the First Affiliated Hospital of Zhengzhou University [Grant No. 71093], and National Key R&D Program of China [Grant No. 2018YFC1002206].

## Conflict of Interest

The authors declare that the research was conducted in the absence of any commercial or financial relationships that could be construed as a potential conflict of interest.

## Publisher's Note

All claims expressed in this article are solely those of the authors and do not necessarily represent those of their affiliated organizations, or those of the publisher, the editors and the reviewers. Any product that may be evaluated in this article, or claim that may be made by its manufacturer, is not guaranteed or endorsed by the publisher.

## Abbreviations

SCA: Sex Chromosome Aneuploidy
NIPT: Noninvasive Prenatal Testing
PPV: Positive Predictive Value
cffDNA: Cell-Free Fetal DNA
MPS: Massively Parallel Sequencing
IPD: Invasive Prenatal Diagnosis
cfDNA: cell-free DNA
MSS: maternal serum screening
TP: true positive
FP: false positive
NA: not available.
